# Supplementary material for: Modeling glioblastoma heterogeneity as a dynamic network of cell states
Source: Mol Syst Biol. 2021 Sep 16;17(9):e10105. doi: 10.15252/msb.202010105 (PMC8444284; doi:10.15252/msb.202010105)
Supplement: Supplementary file 6 — Source Data for Figure 5 [file MSB-17-e10105-s004.zip › Figure5A_sourcedata/GSEA_3071/nosigdb_stateV.GseaPreranked.1622795159954/DALMO_BARCODENUMBER_UNPUB.html]

Details for gene set DALMO\_BARCODENUMBER\_UNPUB[GSEA]

|  || Dataset | state43071 |
| Phenotype | NoPhenotypeAvailable |
| Upregulated in class | na\_pos |
| GeneSet | DALMO\_BARCODENUMBER\_UNPUB |
| Enrichment Score (ES) | 0.57639205 |
| Normalized Enrichment Score (NES) | 3.4184415 |
| Nominal p-value | 0.0 |
| FDR q-value | 0.0 |
| FWER p-Value | 0.0 |
Table: GSEA Results Summary

  

Fig 1: Enrichment plot: DALMO\_BARCODENUMBER\_UNPUB      
 Profile of the Running ES Score & Positions of GeneSet Members on the Rank Ordered List

  

| PROBE | GENE SYMBOL | GENE\_TITLE | RANK IN GENE LIST | RANK METRIC SCORE | RUNNING ES | CORE ENRICHMENT || 1 | HIST1H1B |  |  | 0 | 2.500 | 0.0173 | Yes |
| 2 | HIST1H4C |  |  | 1 | 2.442 | 0.0342 | Yes |
| 3 | ESCO2 |  |  | 3 | 2.193 | 0.0492 | Yes |
| 4 | HMGB2 |  |  | 5 | 2.011 | 0.0629 | Yes |
| 5 | NUSAP1 |  |  | 6 | 2.001 | 0.0767 | Yes |
| 6 | TOP2A |  |  | 14 | 1.842 | 0.0881 | Yes |
| 7 | DHFR |  |  | 18 | 1.795 | 0.1000 | Yes |
| 8 | NCAPG |  |  | 21 | 1.760 | 0.1117 | Yes |
| 9 | H2AFX |  |  | 26 | 1.725 | 0.1229 | Yes |
| 10 | PCLAF |  |  | 27 | 1.720 | 0.1348 | Yes |
| 11 | CENPF |  |  | 28 | 1.705 | 0.1466 | Yes |
| 12 | RRM2 |  |  | 34 | 1.660 | 0.1571 | Yes |
| 13 | ITGA7 |  |  | 37 | 1.644 | 0.1681 | Yes |
| 14 | SGO1 |  |  | 40 | 1.614 | 0.1789 | Yes |
| 15 | MKI67 |  |  | 45 | 1.598 | 0.1891 | Yes |
| 16 | KIF15 |  |  | 52 | 1.568 | 0.1988 | Yes |
| 17 | FOXM1 |  |  | 53 | 1.550 | 0.2096 | Yes |
| 18 | PBK |  |  | 54 | 1.536 | 0.2202 | Yes |
| 19 | GTSE1 |  |  | 66 | 1.491 | 0.2284 | Yes |
| 20 | COL9A3 |  |  | 68 | 1.488 | 0.2385 | Yes |
| 21 | CDK1 |  |  | 69 | 1.488 | 0.2488 | Yes |
| 22 | SMC2 |  |  | 73 | 1.466 | 0.2583 | Yes |
| 23 | SPC25 |  |  | 76 | 1.442 | 0.2679 | Yes |
| 24 | UBE2T |  |  | 79 | 1.432 | 0.2774 | Yes |
| 25 | FANCD2 |  |  | 81 | 1.423 | 0.2871 | Yes |
| 26 | CIP2A |  |  | 86 | 1.410 | 0.2960 | Yes |
| 27 | DEK |  |  | 94 | 1.384 | 0.3043 | Yes |
| 28 | CDCA2 |  |  | 96 | 1.382 | 0.3136 | Yes |
| 29 | KIFC1 |  |  | 113 | 1.338 | 0.3198 | Yes |
| 30 | CENPM |  |  | 114 | 1.337 | 0.3290 | Yes |
| 31 | TACC3 |  |  | 116 | 1.334 | 0.3380 | Yes |
| 32 | CDCA5 |  |  | 123 | 1.326 | 0.3460 | Yes |
| 33 | MIS18BP1 |  |  | 159 | 1.247 | 0.3479 | Yes |
| 34 | CDKN3 |  |  | 163 | 1.239 | 0.3558 | Yes |
| 35 | KIF23 |  |  | 167 | 1.235 | 0.3638 | Yes |
| 36 | NCAPH |  |  | 168 | 1.235 | 0.3724 | Yes |
| 37 | CDCA3 |  |  | 169 | 1.230 | 0.3809 | Yes |
| 38 | KIF4A |  |  | 191 | 1.193 | 0.3850 | Yes |
| 39 | NUF2 |  |  | 192 | 1.189 | 0.3933 | Yes |
| 40 | TCEAL7 |  |  | 200 | 1.171 | 0.4000 | Yes |
| 41 | ASPM |  |  | 207 | 1.163 | 0.4069 | Yes |
| 42 | UBE2C |  |  | 214 | 1.154 | 0.4137 | Yes |
| 43 | FAM210B |  |  | 218 | 1.151 | 0.4211 | Yes |
| 44 | NCAPG2 |  |  | 225 | 1.141 | 0.4278 | Yes |
| 45 | SGO2 |  |  | 244 | 1.126 | 0.4321 | Yes |
| 46 | TPX2 |  |  | 248 | 1.122 | 0.4393 | Yes |
| 47 | BUB1 |  |  | 277 | 1.084 | 0.4413 | Yes |
| 48 | ZBTB20 |  |  | 281 | 1.080 | 0.4482 | Yes |
| 49 | CKAP2L |  |  | 285 | 1.078 | 0.4551 | Yes |
| 50 | BEX1 |  |  | 287 | 1.077 | 0.4623 | Yes |
| 51 | AURKB |  |  | 297 | 1.068 | 0.4680 | Yes |
| 52 | ARHGAP11A |  |  | 303 | 1.064 | 0.4743 | Yes |
| 53 | CCNA2 |  |  | 309 | 1.057 | 0.4807 | Yes |
| 54 | PRR11 |  |  | 317 | 1.048 | 0.4866 | Yes |
| 55 | PIMREG |  |  | 339 | 1.025 | 0.4896 | Yes |
| 56 | CDKN1A |  |  | 363 | 0.993 | 0.4920 | Yes |
| 57 | KIF11 |  |  | 369 | 0.982 | 0.4978 | Yes |
| 58 | PHF19 |  |  | 378 | 0.971 | 0.5029 | Yes |
| 59 | HJURP |  |  | 406 | 0.945 | 0.5042 | Yes |
| 60 | SPC24 |  |  | 414 | 0.938 | 0.5094 | Yes |
| 61 | DLGAP5 |  |  | 418 | 0.936 | 0.5152 | Yes |
| 62 | MAD2L1 |  |  | 421 | 0.932 | 0.5213 | Yes |
| 63 | KPNA2 |  |  | 435 | 0.919 | 0.5251 | Yes |
| 64 | CENPE |  |  | 437 | 0.918 | 0.5313 | Yes |
| 65 | RACGAP1 |  |  | 443 | 0.912 | 0.5366 | Yes |
| 66 | HMGN2 |  |  | 466 | 0.900 | 0.5386 | Yes |
| 67 | POC1A |  |  | 467 | 0.896 | 0.5448 | Yes |
| 68 | PCSK1N |  |  | 476 | 0.887 | 0.5493 | Yes |
| 69 | PLK1 |  |  | 526 | 0.850 | 0.5457 | Yes |
| 70 | BIRC5 |  |  | 541 | 0.842 | 0.5488 | Yes |
| 71 | RALGAPA2 |  |  | 573 | 0.815 | 0.5484 | Yes |
| 72 | HMGB1 |  |  | 583 | 0.810 | 0.5522 | Yes |
| 73 | DIAPH3 |  |  | 585 | 0.808 | 0.5576 | Yes |
| 74 | TK1 |  |  | 591 | 0.806 | 0.5622 | Yes |
| 75 | CENPA |  |  | 627 | 0.786 | 0.5609 | Yes |
| 76 | PIF1 |  |  | 632 | 0.782 | 0.5655 | Yes |
| 77 | SKA3 |  |  | 668 | 0.765 | 0.5640 | Yes |
| 78 | KIF18B |  |  | 698 | 0.747 | 0.5635 | Yes |
| 79 | GRIA2 |  |  | 713 | 0.741 | 0.5659 | Yes |
| 80 | PLK4 |  |  | 720 | 0.739 | 0.5698 | Yes |
| 81 | ANP32E |  |  | 722 | 0.739 | 0.5747 | Yes |
| 82 | TMEM59L |  |  | 768 | 0.715 | 0.5709 | Yes |
| 83 | LONRF2 |  |  | 785 | 0.706 | 0.5727 | Yes |
| 84 | APLP1 |  |  | 792 | 0.704 | 0.5764 | Yes |
| 85 | H2AFZ |  |  | 851 | 0.677 | 0.5698 | No |
| 86 | MDM2 |  |  | 904 | 0.658 | 0.5642 | No |
| 87 | MZT1 |  |  | 917 | 0.652 | 0.5664 | No |
| 88 | H2AFV |  |  | 933 | 0.647 | 0.5679 | No |
| 89 | PTTG1 |  |  | 936 | 0.647 | 0.5720 | No |
| 90 | CENPN |  |  | 953 | 0.638 | 0.5733 | No |
| 91 | CKS2 |  |  | 966 | 0.634 | 0.5754 | No |
| 92 | LRR1 |  |  | 991 | 0.623 | 0.5750 | No |
| 93 | CHPF |  |  | 1021 | 0.615 | 0.5736 | No |
| 94 | DDR1 |  |  | 1064 | 0.603 | 0.5696 | No |
| 95 | SRSF2 |  |  | 1084 | 0.597 | 0.5700 | No |
| 96 | ZMAT3 |  |  | 1129 | 0.585 | 0.5655 | No |
| 97 | IGSF8 |  |  | 1146 | 0.579 | 0.5664 | No |
| 98 | HMGB3 |  |  | 1154 | 0.577 | 0.5690 | No |
| 99 | YPEL3 |  |  | 1159 | 0.575 | 0.5722 | No |
| 100 | ANP32B |  |  | 1187 | 0.566 | 0.5709 | No |
| 101 | PARPBP |  |  | 1210 | 0.560 | 0.5705 | No |
| 102 | VGLL4 |  |  | 1262 | 0.541 | 0.5643 | No |
| 103 | KIF2C |  |  | 1309 | 0.530 | 0.5590 | No |
| 104 | C9orf16 |  |  | 1368 | 0.516 | 0.5513 | No |
| 105 | CCNB2 |  |  | 1373 | 0.513 | 0.5540 | No |
| 106 | SPDL1 |  |  | 1397 | 0.507 | 0.5531 | No |
| 107 | PGRMC1 |  |  | 1498 | 0.480 | 0.5369 | No |
| 108 | CKS1B |  |  | 1502 | 0.479 | 0.5396 | No |
| 109 | PLEKHB1 |  |  | 1520 | 0.477 | 0.5396 | No |
| 110 | HIST1H1A |  |  | 1533 | 0.473 | 0.5406 | No |
| 111 | PCDH9 |  |  | 1592 | 0.461 | 0.5325 | No |
| 112 | TUBB4B |  |  | 1659 | 0.444 | 0.5227 | No |
| 113 | IRX1 |  |  | 1785 | 0.421 | 0.5013 | No |
| 114 | PSAP |  |  | 1855 | 0.410 | 0.4907 | No |
| 115 | DAPL1 |  |  | 1945 | 0.397 | 0.4761 | No |
| 116 | HTRA1 |  |  | 1958 | 0.395 | 0.4765 | No |
| 117 | CENPW |  |  | 1959 | 0.395 | 0.4792 | No |
| 118 | LSM5 |  |  | 1982 | 0.391 | 0.4776 | No |
| 119 | LAMB2 |  |  | 2003 | 0.387 | 0.4764 | No |
| 120 | CHD9 |  |  | 2108 | 0.373 | 0.4587 | No |
| 121 | ANLN |  |  | 2125 | 0.371 | 0.4582 | No |
| 122 | CHST7 |  |  | 2377 | 0.338 | 0.4116 | No |
| 123 | PAICS |  |  | 2403 | 0.334 | 0.4091 | No |
| 124 | HPRT1 |  |  | 2404 | 0.334 | 0.4114 | No |
| 125 | PCDHB9 |  |  | 2439 | 0.330 | 0.4071 | No |
| 126 | CCNB1 |  |  | 2480 | 0.325 | 0.4015 | No |
| 127 | IP6K2 |  |  | 2645 | 0.305 | 0.3717 | No |
| 128 | RRM2B |  |  | 2709 | 0.298 | 0.3615 | No |
| 129 | KIF3C |  |  | 2716 | 0.298 | 0.3624 | No |
| 130 | SEZ6L2 |  |  | 2958 | 0.273 | 0.3173 | No |
| 131 | PLXNB2 |  |  | 3068 | 0.262 | 0.2979 | No |
| 132 | PROS1 |  |  | 3097 | 0.260 | 0.2943 | No |
| 133 | CCT8 |  |  | 3227 | -0.255 | 0.2709 | No |
| 134 | MMP2 |  |  | 3234 | -0.257 | 0.2715 | No |
| 135 | COL18A1 |  |  | 3251 | -0.259 | 0.2702 | No |
| 136 | IDS |  |  | 3288 | -0.264 | 0.2650 | No |
| 137 | ATP6V1G1 |  |  | 3316 | -0.270 | 0.2616 | No |
| 138 | GDF15 |  |  | 3713 | -0.333 | 0.1868 | No |
| 139 | CYSTM1 |  |  | 3782 | -0.346 | 0.1759 | No |
| 140 | PCMT1 |  |  | 3788 | -0.347 | 0.1774 | No |
| 141 | RPS27L |  |  | 3927 | -0.373 | 0.1531 | No |
| 142 | CCT5 |  |  | 3958 | -0.379 | 0.1499 | No |
| 143 | GSTM3 |  |  | 3966 | -0.381 | 0.1511 | No |
| 144 | SNRPF |  |  | 4037 | -0.397 | 0.1402 | No |
| 145 | TRIM22 |  |  | 4138 | -0.419 | 0.1237 | No |
| 146 | LDHA |  |  | 4145 | -0.421 | 0.1254 | No |
| 147 | ITGAV |  |  | 4207 | -0.442 | 0.1166 | No |
| 148 | ABCA5 |  |  | 4293 | -0.471 | 0.1033 | No |
| 149 | RPL35 |  |  | 4365 | -0.490 | 0.0929 | No |
| 150 | GADD45A |  |  | 4399 | -0.500 | 0.0899 | No |
| 151 | IGFBP7 |  |  | 4622 | -0.604 | 0.0508 | No |
| 152 | PLTP |  |  | 4682 | -0.634 | 0.0437 | No |
| 153 | SMS |  |  | 4842 | -0.750 | 0.0180 | No |
| 154 | HMGA1 |  |  | 4962 | -0.878 | 0.0009 | No |
| 155 | NUDT14 |  |  | 4996 | -0.926 | 0.0008 | No |
| 156 | S100A10 |  |  | 5011 | -0.954 | 0.0047 | No |
| 157 | SNHG8 |  |  | 5105 | -1.165 | -0.0053 | No |
| 158 | PHLDA3 |  |  | 5150 | -1.320 | -0.0048 | No |
| 159 | VPS13C |  |  | 5169 | -1.412 | 0.0015 | No |
| 160 | S100A6 |  |  | 5183 | -1.473 | 0.0092 | No |
| 161 | LRP1 |  |  | 5234 | -1.801 | 0.0119 | No |
Table: GSEA details [plain text format]

  

Fig 2: DALMO\_BARCODENUMBER\_UNPUB: Random ES distribution      
 Gene set null distribution of ES for **DALMO\_BARCODENUMBER\_UNPUB**
